# Dental Education

**Published:** 1882-11

**Authors:** 


### EDITORIAL, ETC.

---

*Dental Education* —The winter or regular Sessions of the Dental Schools throughout the country have now fairly commenced, and are no doubt being conducted with the characteristic energy which distinguishes such institutions. While, owing to the large, number of such schools, some are struggling for existence, the attendance at the more advanced institutions is large and everything is progressing in a satisfactory and harmonious manner.

Since the issue of the October No. of this JOURNAL the *unprecedented* success of the Dental Department of the University of Maryland has continued until at this time (the first part of the month of November) the number of matriculants is considerably over sixty. Such an auspicious opening of the *first* session of this University Department is not only gratifying but beyond expectation, and will be received by its friends as an indication of what the future may develop, and also an evidence that the dental profession generally regards a reputable University as the proper place for the instruction of dental students.

